# Preparation and Evaluation of the Antibacterial Effect of Chitosan Nanoparticles Containing Ginger Extract Tailored by Central Composite Design

**DOI:** 10.34172/apb.2021.073

**Published:** 2020-09-08

**Authors:** Ali Farmoudeh, Aynaz Shokoohi, Pedram Ebrahimnejad

**Affiliations:** ^1^Department of Pharmaceutics, Faculty of Pharmacy, Mazandaran University of Medical Sciences, Sari, Iran.; ^2^Pharmaceutical Sciences Research Center, Mazandaran University of Medical Sciences, Ramsar International Branch, Ramsar, Iran.

**Keywords:** Ginger root extract, Chitosan, Nanoparticles, MIC, Drug delivery

## Abstract

***Purpose:*** The ginger root extract has shown remarkable antimicrobial effects. Nanocarriers based on biodegradable polymers (like chitosan) are promising drug delivery vehicles for antibacterial compounds. In this study, aqueous and methanolic extracts of ginger root were prepared, loaded on chitosan nanoparticles (NPs), and their antimicrobial effects were investigated.

***Methods:*** The NPs were prepared using the ionic gelation technique. The central composite design model was employed to optimize the formulation variables and achieve the minimum particle size and maximum zeta potential. The total phenol content of the powdered extracts was determined. The antimicrobial activity of the NPs was evaluated by the determination of minimum inhibitory concentration (MIC) and minimum bactericidal concentration (MBC).

***Results:*** The optimum size of NPs containing methanolic or aqueous extract were 188.3 and 154.7 nm, with a zeta potential of 29.1 and 32.1 mv, and entrapment efficiency percent (E.E.%) of 61.57±3.12% and 44.26±2.57%, respectively. Transmission electronic microscopy images confirmed the spherical particles in the low nanometer range. The phenol content of methanol extract was higher than the aqueous one (60.216 ± 1.83 and 39.835 ± 1.72 mg gallic acid equivalent/100 g), respectively). According to the results of the MIC and MBC, methanol extract NPs showed more potent antimicrobial effects, which seems to be associated with higher concentrations of phenolic compounds. The FTIR spectrophotometry showed no chemical interaction between the extracts and other ingredients.

***Conclusion:*** The results demonstrated that current NPs significantly increased the antibacterial effects of ginger extracts and could be selected for further evaluation.

## Introduction

Antimicrobial resistance has become a serious global threat in recent years. In this situation, researchers are widely working to find new treatments. Synthesis of a new effective antibiotic is very time consuming and expensive. For this reason, many new studies have focused on natural compounds and new drug delivery systems.^1,2^ Plant extracts are a natural source for antibiotics that have attracted particular attention due to low production cost and high biocompatibility. Among the herbs, ginger (*Zingiber officinale*) has shown significant antimicrobial effects related to its phenolic compounds. The spicy taste of ginger root is due to the presence of gingerol, which has a phenolic structure.^3^ Ginger extracts show antibacterial activity against the pathogens of *Staphylococcus aureus* and *Escherichia coli.*^4,5^ There are also many reports of anti-inflammatory and anticancer effects of this plant extract.^6-8^ Designing novel drug delivery systems is another way to increase the effectiveness of existing drugs. Recent studies have shown that the use of colloidal systems to transport antimicrobial compounds enhances their therapeutic effects.^9-11^ Nanoparticles (NPs) protect the drug against environmental factors and bacterial enzymes.^12^ Also, by binding the appropriate compounds to the surface of the NPs, they will find the ability to target microorganisms that enhance the efficiency of treatment.^13^ Chitosan (CHI) is one of the biocompatible and biodegradable polymers widely used in pharmaceutical formulations. In an acidic medium, the surface charge of this polymer is positive, and this property is used to formulate targeted NPs.^14,15^ Studies have shown that chitosan binding to lipopolysaccharides on the bacterial surface disrupts the order of the bacterial membrane and causes the plasma to leak out.^16^ It also increases the chances of drug penetration into the bacterial cytoplasm.^17^ In this study, aqueous and methanolic extracts were obtained from ginger root individually, and then CHI NPs containing each extract were prepared. The optimization of formulations was performed using the central composite design statistical model. The antibacterial effects of the optimal formulations were investigated.

## Materials and Methods

### Materials

Chitosan was purchased from Solarbio chemicals (China), Tripolyphosphate (TPP), Acetic acid, Sodium hydroxide (NaOH), and Potassium dihydrogen phosphate from Merck KGaA (Germany). Fresh deionized water was obtained from the Human Ultra-Pure System (Human Corp, Korea).

### Preparation of ginger root extracts

Dried ginger root was grounded to pass 160 mesh sieves. Powdered root samples (20 g) were extracted with 250 mL of 99% methanol or distilled water. Then each container was shaken for 72 hours at room temperature. After filtration, solvents were evaporated using a rotary evaporator and freeze drier. Dried extracts were then weighed and stored at 4°C.

### Total phenol content evaluation

Total phenolic contents in ginger root extracts were determined using the Folin-Ciocalteu assay method. Briefly, Folin-Ciocalteu reagent (0.5 mL) and 7.5 mL distilled water were added in 50 mg of plant extracts. After 10 minutes of incubation at room temperature, 1.5 ml of sodium carbonate (20%) was added to the mixture and heated for 20 minutes (at 40°C). UV absorbance was measured at 755 nm. The result was expressed as gallic acid equivalents mg in 100 g of powdered root extraction briefly expressed as GAE (mg)/100 g.

### Preparation ofNPs

100 mg of CHI was immersed in 100 ml of acetic acid (1%) solution for 24 hours to be completely hydrated and then wholly dissolved to obtain a uniform solvent (1 mg/mL solution of CHI). The aqueous extract of ginger root (50 mg) was added to the polymer solution and dispersed in the medium by stirring for 1 hour. Next, a 1mg/mL solution of TPP was prepared in deionized distilled water. In different formulations, various volumes of TPP solution were added to the drug-containing polymer solution (Table 1). The formulation was then stirred for 2 hours to complete the charge interference process and the formation of the NPs. Subsequently, the formulation was placed in an ultrasonic bath at different times to reduce the size of dispersed particles.^18,19^ In the process of preparing NPs of methanolic extract, 50 mg of the extract was dissolved in 2 mL of propylene glycol (as a co-solvent), then the dispersion was added to the CHI solution.

**Table 1:** Experimental variables and observed responses for CCD

**Run**	**Block**	**Variables**		**Responses**		
		**X1**	**X2**	**Y1**	**Y2**	**Y3**
		**Chitosan/ TPP** **(concentration ratio)**	**Sonication time** **(min)**	**Zeta potential** **(mv)**	**Size** **(nm)**	**Polydispersity index**
F1	Aqueous extract	1.00	30.00	11.267	661.967	0.747
F2	Aqueous extract	3.00	18.00	23.900	292.267	0.321
F3	Aqueous extract	3.00	18.00	25.667	308.233	0.279
F4	Aqueous extract	3.00	18.00	21.167	267.900	0.365
F5	Aqueous extract	1.00	6.00	4.267	798.967	0.929
F6	Aqueous extract	5.00	30.00	33.167	142.467	0.213
F7	Aqueous extract	5.00	6.00	24.033	304.333	0.587
F8	Methanol extract	3.00	1.03	18.500	454.633	0.701
F9	Methanol extract	3.00	18.00	23.433	368.667	0.353
F10	Methanol extract	5.83	18.00	36.893	171.100	0.281
F11	Methanol extract	3.00	18.00	20.240	300.133	0.282
F12	Methanol extract	3.00	18.00	21.767	341.667	0.350
F13	Methanol extract	3.00	34.97	26.867	223.933	0.228
F14	Methanol extract	0.17	18.00	5.067	905.667	0.948

Data are shown as mean ± standard deviation, n = 3.

### Central composite design (CCD)

The CCD statistical model was used to optimize the variables in this study. The volume ratio of CHI to TPP (Y1) and sonic duration (Y2) was selected as independent variables, and three levels of minimum (-1), average (0), and the maximum (+1) were considered for each. The formulations were divided into two blocks, namely aqueous extract and methanolic extract. Fourteen experimental formulations were proposed by the design expert 7.0.0 software (Table 1). The formulas were prepared in the laboratory, and the values of particle size, zeta potential, and polydispersity index were recorded as dependent variables. The minimum size and the maximum zeta potential values were considered desirable properties, and the optimal formulation predicted by CCD was prepared in the laboratory, and its antimicrobial effects were evaluated.

### Evaluation of nanoparticles

#### Particle size, polydispersity index, and zeta potential of NPs

Mean particle size and polydispersity index determination of each prepared formulation were performed by a laser light scattering technique using Zetasizer NANO (Malvern, Worcestershire, United Kingdom). The amount of entrapped phenolic compounds in the NPs was evaluated using the centrifugation method. The final colloidal dispersion was centrifuged at 20 000 rpm at 4^o^C for 45 minutes (SIGMA 3-30KS centrifuge, Germany). Then, the supernatant was quantified using the Folin-Ciocalteu assay method. The following equation was used to calculate the entrapment efficiency percent (E.E.%):

$$EE\%=\frac{Encapsulated\;drugs\;weight}{Total\;drug\;weight}\times 100$$

Samples were diluted (1: 5) with deionized water before each performance to keep the scattering intensity between 150-350 kcps. Three replications were done, and mean values were recorded. The zeta potential of NPs was measured at 25 °C using the same instrument and protocol.^10,20,21^

#### FTIR spectroscopy

The possible interaction between formulation ingredients was investigated by FT-IR infrared spectroscopy (Biochrom Ltd., Cambridge CB4 OF England). Samples of pure CHI, TPP, powdered extracts (aqueous and methanolic), and freeze-dried nanosuspensions were scanned in the range of 4000–400 cm^-1^ at a resolution of 1 cm^-1^. The dried sample was grounded with KBr and compressed into a suitable-size disk (13 mm), then placed inside the apparatus for IR spectroscopy.^22^

#### Transmission electron microscopy (TEM)

The morphological state of optimized CHI NPs was evaluated using an EM 208S transmission electron microscope (Philips, the Netherlands) operating at 100 kV. In brief, a drop of the nanosuspension was placed on a copper grid coated with carbon. The sample was dried at room temperature and prepared according to the manufacturer’s protocol for microscopic examination.

### Assays for antibacterial activity

#### Determination of minimum inhibitory concentration (MIC) by broth microdilution method

The MIC test was determined according to the Clinical and Laboratory Standards Institute (CLSI) broth microdilution method. Suspension of aqueous and methanolic ginger root extracts, nanosuspension of each extract (500 μg/mL), and free drug CHI nanosuspension (500 μg/mL) were examined to evaluate MIC values (Table 2). Various concentrations of samples were prepared in Mueller-Hinton broth medium (256, 128, 64, 32, 16, 8, 4, 2, 1, 0.5, and 0.25 μg/mL). A volume of 180 mL of the prepared concentrations was added to each well, followed by inoculation with 10 µL of microbial suspension (final bacterial density: 5×10^5^ CFU/mL). The microplates were incubated at 35°C for 18 hours, then the degree of turbidity (related to the growth of the microorganisms) was investigated.

**Table 2:** Microdilution broth MIC and MBC values of prepared formulations against some pathogenic bacteria (µg/mL)

	**Aqueous extract**		**Methanolic extract**		**Nanosuspension of aqueous extract**		**Nanosuspension of methanolic extract**		**Drug-free nanosuspension**	
	MIC	MBC	MIC	MBC	MIC	MBC	MIC	MBC	MIC	MBC
*S. aureus*	62.5	250	31.25	125	62.5	250	15.62	62.48	125	-
*E. faecalis*	62.5	500	62.5	500	62.5	250	62.5	250	125	-
*P. aeruginosa*	62.5	250	62.5	500	31.25	125	62.5	500	250	-
*A. baumannii*	62.5	500	62.5	500	62.5	500	62.5	500	250	-
*E. coli*	62.5	250	62.5	250	62.5	250	15.62	62.48	125	-
*K. pneumoniae*	62.5	250	62.5	500	62.5	250	62.5	250	125	-
*P. mirabilis*	62.5	500	62.5	250	62.5	250	62.5	250	125	-

#### Determination of minimum bactericidal concentration (MBC)

In this test, dilution represented the MIC, and three wells before (those above the MIC), were transferred to a plate containing Blood Agar and incubated at 37 °C for 24 hours. Then the lowest broth dilution of the extract that prevented the growth of 99.5% of the bacterial colonies was reported as MBC.^23-25^

### Statistical analysis of data

Statistical analysis was performed using a T-test to compare the means between two groups and ANOVA for three or more. The tests were considered significant at a *P* value < 0.05. The ANOVA analysis was followed by Student-Newman-Keuls (SNK) test to find the group that was significantly different from the others.

## Results and Discussion

### Characteristics ofNPs containing aqueous or methanol extract

The CCD statistical model provided 14 formulations to investigate the influence of independent variables on formulation properties. Each formulation was prepared, and the mean size, zeta potential, and particle size distribution were recorded for further investigation. According to Table 1, the particle sizes ranged from 142.367 ± 9.76 to 905.667 ± 19.69. The following equation shows the effect of each independent variable on the particle size:

Size = +327.05 − 256.62× A − 78.14× B +1 20.41× A^2^

**Figure 1:**
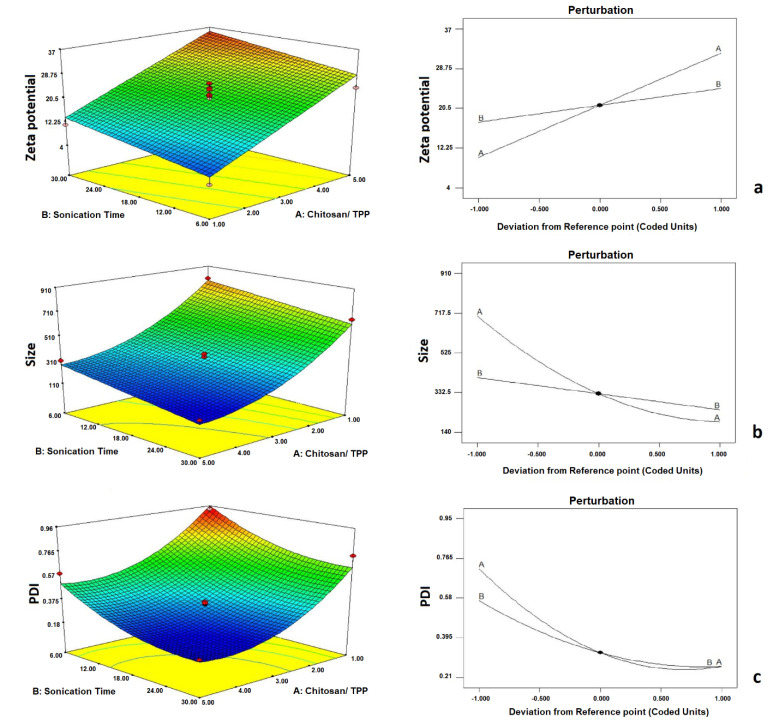
Perturbation plot and response surface 3D plot for the effect of independent variables on zeta potential (ZP) (a), Size (b), and Polydispersity index (c).

According to the presented coefficients, CHI/TPP (A) and sonication time (B) had a significant effect on particle size (*P*  <  0.05). According to Figure 1a, by increasing CHI/ TPP and sonication time, the particle size was decreased significantly (0.05). It should be noted that the electric repulsion between the particles caused by surface charge also causes the colloidal dispersion to stabilize. As the amount of anion in the surface charge environment increases, the CHI filaments accumulate and form larger particles.^24,25^ On the other hand, if the anion content is low in the medium, there are not enough ionic bonds to bend the polymer and form small NPs. Results showed that the particle size distribution varied from 0.213 ± 0.03 to 0.947± 0.05. The effect of each independent variable on the size distribution was calculated by mathematical equation 2.

PDI = +0.32 − 0.23× A − 0.15× B + 0.16× A^2^ + 0.090× B^2^

Due to the equation coefficients, by increasing CHI/ TPP and sonication time, particle size dispersion reduced, and more uniform particles were obtained (*P* <  0.05). Figure 1b shows the effect of the independent variables on the particle size distribution. The absolute value of zeta potential was in the range of 4.266±1.13 mV to 36.893±3.15 mV. The relationship between the variables and zeta potential is shown in the following equation:

Zeta potential = +21.16 +1 0.83× A + 3.50× B

According to the coefficients of the equation, by increasing the ratio of CHI to TPP, the surface charge was significantly increased, predicting the long-term stability of the colloidal dispersions (*P*  <  0.05). Due to the positive zeta potential number, the presence of a high ratio of CHI/ TPP in the formulations has resulted in the positive surface charge of this polymer at the particle surface.^12,26^ The results also showed that by increasing sonication duration, ZP was significantly improved (*P*  <  0.05) (Figure 1c).

### Determination of optimal formulation

**Figure 2:**
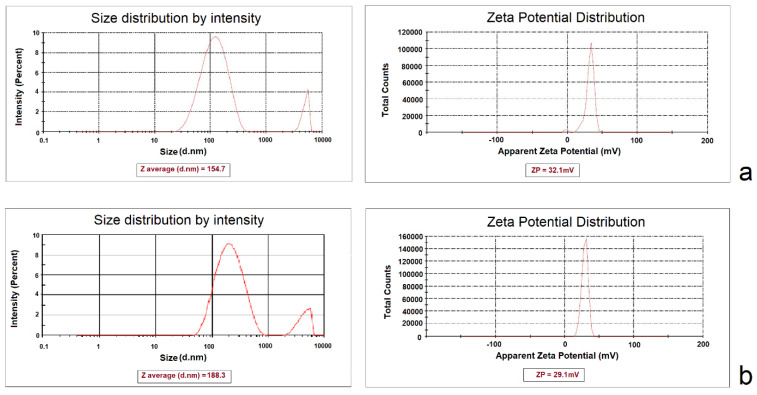
Observed responses values for predicted optimal formulation (a: nanoparticles of aqueous extract, b: nanoparticles of methanolic extract).

**Figure 3:**
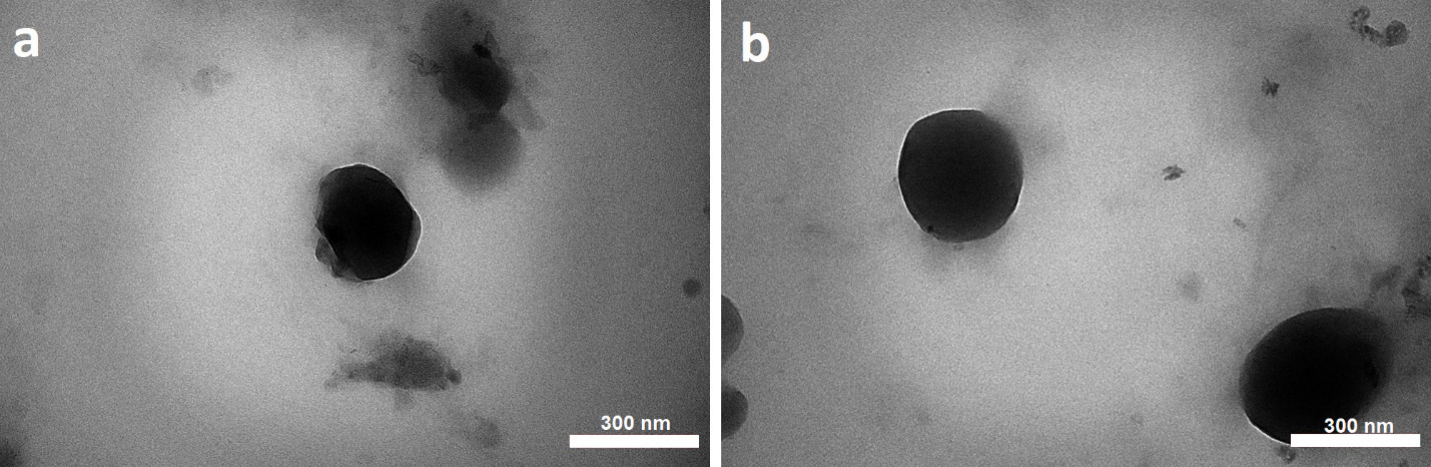
TEM images of nanoparticles of aqueous extract (a) and methanolic extract (b).

CCD is an experimental design used to optimize a response (dependent variable), which is influenced by several independent variables. In this study, reducing the size of NPs and increasing surface electric charges were considered as desirable properties for the determination of optimum formulation. The 5:1 concentration ratio of CHI/ TPP and a sonication time of 21.34 minutes were calculated as the optimum independent variables to optimize the dependent variables simultaneously. Predicted size and ZP were 157.422±1.12 nm and 33.49±0.956 mV, respectively. The predicted optimum formulations of aqueous and methanolic extracts were prepared in the laboratory and evaluated for particle size and ZP, which are close to the predicted values (Figure 2). The E.E% of phenolic compounds in optimized NPs of aqueous and alcoholic extracts were calculated to be 44.26±2.57% and 61.57±3.12%, respectively. The morphology of the optimized NPs was investigated by TEM microscopy. The microscopic images showed the presence of spherical particles in the low nanometer range (Figure 3).

### FTIR spectroscopy

**Figure 4:**
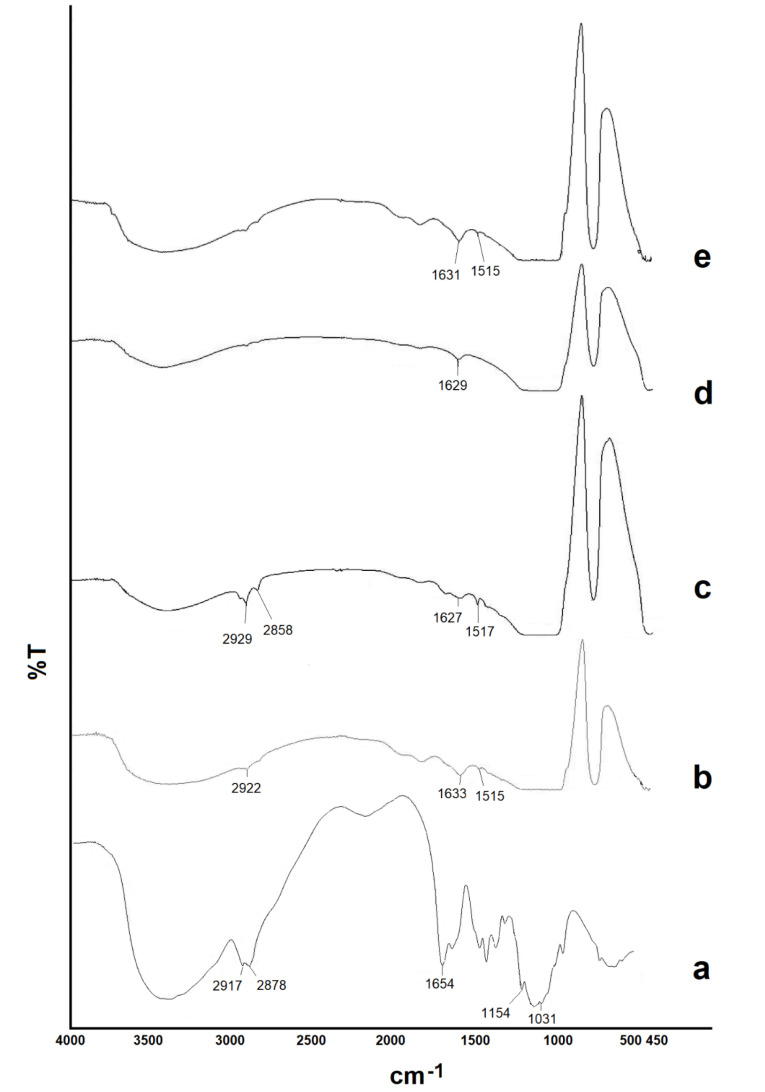
The FTIR spectra of chitosan (a), powdered aqueous (b) and methanolic (c) extracts, and freeze-dried nanoparticles of aqueous (d) and methanolic (e) extracts.

The FTIR spectra of CHI, powdered aqueous and methanolic extracts and freeze-dried NPs are shown in Figure 4. IR spectrum of the aqueous extract showed a large, broad peak at 3000-3600 cm^−1^ associated with stretching vibrations of the OH group of polysaccharides in the extract. The characteristic peaks as the carboxyl group at 2922 cm^-1^, the aromatic ring (C=C) at 1515 cm^-1^, the alkene CH group at 1633 cm^-1^. The spectrum of the NPs showed the characteristic peaks of the aqueous extract, indicated no chemical interaction between formulation components. The spectrum of the alcoholic extract showed characteristic peaks as OH stretching (a large, broad peak in the range of 3000–3600 cm^−1^) The carboxyl group at 2929 cm^-1^ and 2858 cm^-1^, alkene CH group at 1627 cm^-1^, and the aromatic ring (C=C) at 1517 cm^-1^.^27^ The aromatic ring peak in the FTIR spectrum of the alcoholic extract appeared prominently, indicating a higher accumulation of phenolic compounds (flavonoids and phenolic acids) in the extract.^28^ The characteristic peaks of phenolic compounds were determined in the IR spectrum of the NPs. In the FTIR spectrum of the CHI, the major peaks are as follows: A broad peak between 3000-3700 cm^-1^ indicates the presence of the OH (stretching). CH stretching of CH_2_OH at 2878 cm^-1^ and 2917 cm^-1^, the type 1 amide group (C-N-H) at 1654 cm^-1^, the peaks appearing at 1154 cm^-1^ and 1031 cm^-1^ related to the ether group (C-O-C).^29,30^ In the FTIR spectrum of NPs, the presence of a broad peak of the hydroxyl group indicated the formation of a hydrogen bond between the CHI functional groups and TPP.^30,31^

### The results of the MIC by the broth microdilution method

The MIC of aqueous extract of ginger root was observed in the range from 62.50 µg/mL to 125 µg/mL, and the extracts showed activity against *P. aeruginosa* and *E. coli*. The MIC of methanolic extracts ranged between 31.250 µg/mL to 104.167 µg/mL, and the extract exhibited remarkable antimicrobial activity against *S. aureus and E. coli* (31.25 and 52.08 µg/mL respectively, Table 2). The results show that the antibacterial effects of the NPs were significantly greater than the extract (*P*  < 0.05). It should be noted that the drug-free NPs had no significant antibacterial effects (MIC = 166.67-250 µg/mL). Total phenol content test results showed that the amount of phenolic content in alcoholic extract was higher than the aqueous one (60.216 ± 1.83 and 39.835 ± 1.72, respectively). Due to the higher concentration of phenolic substances in the alcoholic extract and the results of the MIC test, the antimicrobial effects can be attributed to these compounds. The minor antimicrobial effects observed in drug-free NPs are related to the presence of CHI in the formulation. The effects of this polymer against microorganisms have been shown in previous studies.^32,33^

### The results of the MBC by the broth microdilution method

The bactericidal activity of NPs of the aqueous extract against the number of pathogenic bacteria was determined using the MBC test. It should be noted that MBC is the lowest concentration of a drug required to terminate over 99% of the bacteria being tested. The bactericidal activity of aqueous and alcoholic extracts against the bacteria studied is shown in Table 2. According to the MBC test, the aqueous extract of the ginger root had a bactericidal effect against *S. aureus* and *S. aeruginosa*, and the alcoholic extract was effective against *E. coli*. These findings agree with the results of previous studies.^34^ The results indicated that the antimicrobial effect of NPs was significantly enhanced compared to the extracts (*P*  < 0.05). The lowest MBC values were found for the NPs of alcoholic extract, which indicates its high efficiency in killing the pathogenic bacteria.^35^

## Conclusion

In this study, CHI NPs were used to load ginger extract. The NPs were prepared using the ionic gelation technique, and CCD statistical design was employed to prepare NPs with optimum size and ZP. Then, the optimized formulations of aqueous and methanolic extracts were prepared in the laboratory, and their antimicrobial effects were evaluated by MIC and MBC tests. The aqueous and methanolic extracts of ginger root showed antimicrobial effects against some of the studied bacteria, and these effects were significantly enhanced in the NPs of the extracts. Total phenol content test results revealed that the amount of phenolic content in methanol extract was higher than the aqueous one. According to the results of the MIC and MBC, NPs of methanol extract showed more potent antimicrobial effects, which seems to be associated with higher concentrations of phenolic compounds.

In this study, chitosan, a biocompatible compound, was used to prepare a colloidal drug delivery system. The properties of NPs were optimized successfully, and the optimum formulations increased the effectiveness of the ginger root extracts *in vitro*.

## Ethical Issues

This project has been approved by the Ethics Committee, with code number MAZUMS.REC.1398.3123.

## Conflict of Interest

The authors declare no conflicts of interest.

## Acknowledgments

This work was supported by the research council of Mazandaran University of Medical Sciences [grant number 3123].
